# Sturge–Weber syndrome Type I: a rare case report

**DOI:** 10.1097/MS9.0000000000003711

**Published:** 2025-08-08

**Authors:** Batoul Hendieh, Firas Khana, Sevin Ibrahim, Abeer Alsweid, Abdulkader Habash, Alae Kayyali, Bushra Salem, Mouhammed Ali Serio

**Affiliations:** a Department of Pediatrics, Faculty of Medicine, University of Aleppo, Aleppo University Hospital (AUH), Aleppo, Syria; b Department of Medical Imaging and Diagnostic Radiology, Faculty of Medicine, University of Aleppo, Aleppo University Hospital (AUH), Aleppo, Syria

**Keywords:** case report, diffuse choroidal hemangioma, magnetic resonance imaging, stroke-like episodes, Sturge–Weber syndrome

## Abstract

**Introduction and importance:**

To document a rare case of Sturge–Weber syndrome (SWS) Type I with acute neurological symptoms.

**Case presentation:**

An 11-year-old boy, previously diagnosed with Sturge–Weber syndrome (SWS) Type I, presented to the emergency department with acute neurological symptoms that included vomiting, headaches, left-sided hemiparesis, and right-sided deviation of the labial commissure.

**Clinical discussion:**

Sturge–Weber syndrome (SWS) is a rare neurocutaneous disorder characterized by facial port-wine stains, leptomeningeal angiomas, and ocular involvement. Our case presented with preserved cognition despite extensive temporal lobe angiomatosis, contrasting the typical presentation of seizures and developmental delay. The patient then experienced stroke-like episodes from fragile leptomeningeal vasculature. With anticonvulsants and low-dose aspirin therapy, the patient achieved full neurological recovery within the first 3 months and maintained stability during 2 years of follow-up.

**Conclusion:**

This case underscores the diverse clinical spectrum of Sturge–Weber syndrome and emphasizes the crucial role of imaging in achieving an accurate diagnosis. Early identification and treatment are essential to prevent further complications and optimize patient outcomes.

## Introduction and importance

Sturge–Weber syndrome (SWS) is a rare neurocutaneous disorder characterized by port-wine stains, leptomeningeal angiomas, and ocular complications such as glaucoma. Patients typically present with seizures, hemiparesis and developmental delay^[1]^.

This manuscript presents a case of Sturge–Weber syndrome (SWS) Type I with preserved cognitive function and unique radiological findings. It further highlights the variation in both clinical and raidological presentations of SWS.

This case report has been reported in line with the CARE criteria^[2]^ and the TITAN guideline checklist 2025^[3]^.

## Case presentation

### History

A previously healthy 11-year-old Caucasian boy presented to the emergency department with a sudden-onset headache accompanied by recurrent vomiting for several hours. He also developed neck stiffness, a positive Brudzinski sign, left-sided hemiparesis, and right-sided deviation of the oral commissure while speaking.

**HIGHLIGHTS**

- The most common vascular malformation in SWS is the cutaneous port-wine stain.
- Stroke-like episodes can occur from fragile leptomeningeal vasculature.
- Cognition may be preserved despite extensive angiomatosis.
- Intraocular pressure may remain normal despite diffuse choroidal hemangiomas.
- Low-dose aspirin therapy helps prevent stroke-like episodes in SWS.

There were no complications noted at birth, no history of perinatal asphyxia, and there was no need for neonatal resuscitation. He also achieved developmental milestones within the expected time frames and his gross and fine motor, language and social skills were appropriate for his age. There was no family history of genetic or neurological disorders. A third-degree consanguinity between the parents was noted.

### Examination

**Figure 1.**
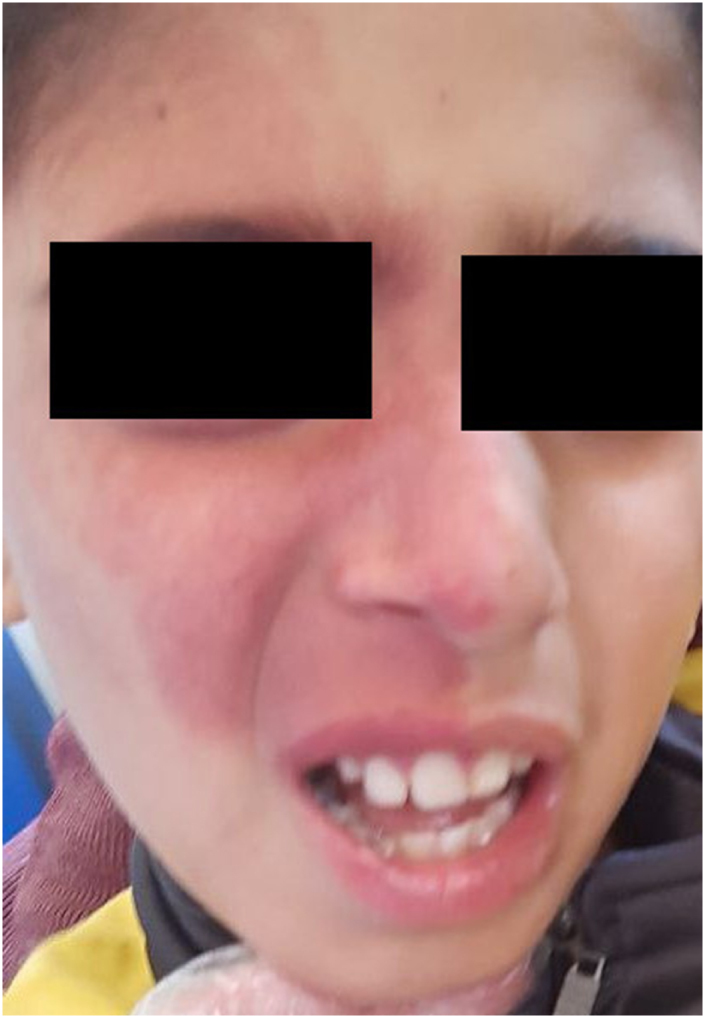
Patient with port-wine stain along the distribution of the first and second divisions of the right trigeminal nerve.

Upon examination, he was conscious but disoriented to time and place, with bilaterally reactive and equal pupils. A port-wine stain, that has been present since birth, was observed on the right half of the face, in the distribution of the first and second divisions of the right trigeminal nerve as shown in Figure 1.

Cranial nerve examination was unremarkable, with the exception of a notable left central facial palsy. The patient also exhibited nuchal rigidity. Motor examination revealed left-sided hemiparesis, with muscle strength graded 2/6 in the left upper and lower limbs, compared to the right side. Chest auscultation and cardiac examination were normal. Abdominal ultrasonography showed no evidence of organomegaly.

### Imaging findings

**Figure 2.**
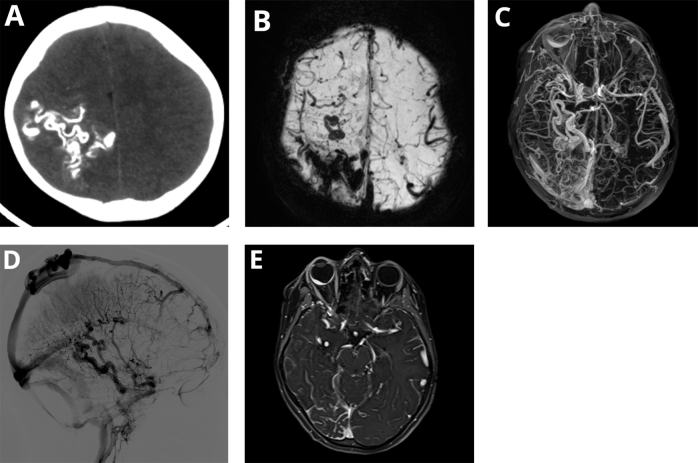
Imaging findings. (A) Axial plane of a noncontrast-enhanced computed tomography of the head showing hyperdense cortical and subcortical tram-track like calcifications in the right parietal and occipital lobes. (B) Axial plane of a susceptibility weighted image (SWI) using minimum intensity projection (MinIP) showing gyriform signal dropouts in the right parietal and occipital lobes. (C) Axial view of a contrast-enhanced fat-suppressed three-dimensional T1-weighted gradient-recalled echo image using maximum intensity projection (MIP) illustrating the large leptomeningeal angioma draining into the straight sinus and extending to the right temporal lobe with a prominent right choroid plexus. (D) Lateral view of a digital subtraction venography (DSV) confirming the extension and drainage of the leptomeningeal angioma. (E) Axial plane of a contrast-enhanced fat-suppressed three-dimensional T1-weighted gradient-recalled echo image showing marked homogenous enhancement of the diffuse right choroidal hemangioma.

The patient was admitted to the intensive care unit for close monitoring. During hospitalization, he experienced two focal tonic-clonic seizures involving the left side of his body, along with persistent rightward deviation of the oral commissure. It was then followed by a generalized tonic-clonic seizure. He was started on intravenous diazepam, then intravenous phenytoin was promptly administered, leading to the resolution of the seizure. A noncontrast-enhanced computed tomography of the head demonstrates hyperdense cortical and subcortical tram-track calcifications in the right parietal and occipital lobes as seen in Figure 2A with relative increase in the thickness of the calvarium over these regions. These serpentine regions appear as signal dropouts on T2*-weighted gradient-recalled echo images and susceptibility weighted images (SWI) produced on a successive magnetic resonance scan of the brain as illustrated in Figure 2B due to calcium and iron deposition. A leptomeningeal angioma was noted in the right parietal and occipital lobes and appears to be extending to the right temporal lobe and draining to the straight sinus, and it appears to be mostly venous in origin as shown in the contrast-enhanced T1-weighted gradient-recalled echo images in Figure 2C and as later confirmed via digital subtraction venography (DSV), which additionally showed a prominent right choroid plexus depicted in Figure 2D. A diffuse right choroidal hemangioma measuring 16.5 × 5.2 mm appearing as a homogenous enhancing convexity post contrast is shown in Figure 2E.

**Figure 3.**
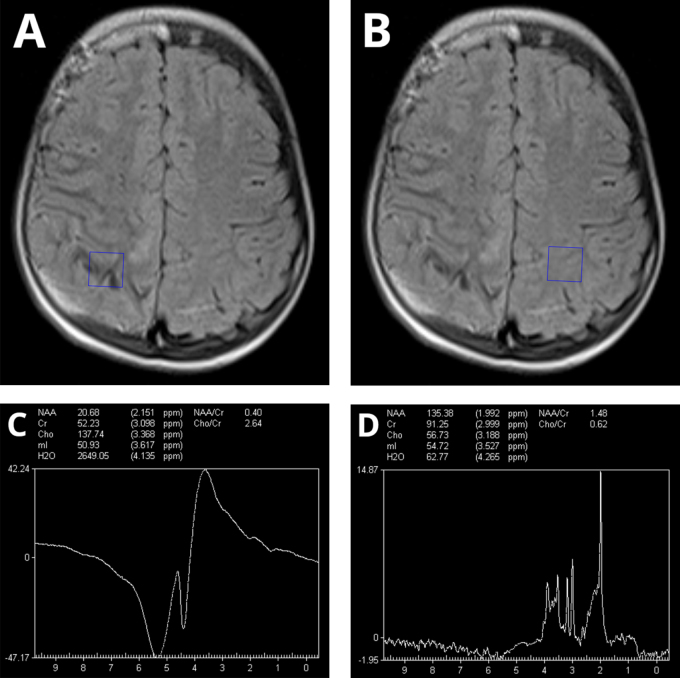
Magnetic resonance spectroscopy findings. (A, B) Localization of voxels at the grey-white matter junction ipsilateral and contralateral to the side of the leptomeningeal angioma, respectively. (C) Ipsilateral spectrum shows decreased *N*-acetyl-aspartate (NAA) and *N*-acetyl-aspartate/creatine (NAA/Cr) ratio and increased choline (Cho) and choline/creatine (Cho/Cr) ratio despite potential signal contamination even when placing saturation bands. (D) Contralateral spectrum also shows a decrease in NAA with a slight decrease in NAA/Cr ratio without a notable increase in Cho and Cho/Cr ratio.

*In vivo* proton magnetic resonance spectroscopy (^1^H-MRS) was performed using single-voxel spectroscopy (SVS) from the grey-white matter junction both ipsilateral and contralateral to the leptomeningeal angioma as illustrated in Figure 3A and B, respectively. Resulting spectra showed decreased *N*-acetyl-aspartate (NAA) and *N*-acetyl-aspartate/creatine (NAA/Cr) ratio and increased choline (Cho) and choline/creatine (Cho/Cr) ratio in the side of the leptomeningeal angioma as seen in Figure 3C, D despite potential signal contamination even when saturation bands were used. This correlates with the clinical findings and potentially reflects ongoing disease activity and tissue reorganization within the affected region.

### Further management

On the second day of admission, the patient developed a fever of 40 °C. Further investigations including a fundoscopy were carried out and showed no evidence of optic disc edema. Comprehensive blood work yielded negative results with the exception of mild anemia. A lumbar puncture revealed 100 RBCs per microliter in the cerebrospinal fluid with no white blood cells, normal protein and high sugar consistent with a possible subarachnoid microhemorrhage. Oral carbamazepine was initiated as an anticonvulsant the following day, while phenytoin was gradually withdrawn and low-dose prophylactic aspirin therapy was administered to prevent future stroke-like episodes. Fever spikes were managed with systemic antipyretics. The nuchal rigidity subsided with treatment progression; however, the left-sided hemiparesis and facial palsy persisted.

The patient was discharged and showed complete recovery of the left-sided hemiparesis and facial nerve palsy within the first 3 months, and maintained stability during 2 years of follow-up. There was no noticeable weakness in the left upper and lower limbs, and muscle strength was within normal limits. Additionally, the patient exhibited normal cranial nerve function and displayed no neurological deficits.

## Discussion

Sturge–Weber syndrome (SWS) is a rare congenital neurocutaneous disorder characterized by a triad of cutaneous, ocular and neurological manifestations. It arises from a somatic mutation in the GNAQ gene that leads to facial capillary malformations (also known as port-wine stains) and associated capillary-venous angiomas affecting the brain and eye. The most common vascular malformation is the cutaneous port-wine stain, occurring in approximately 0.3% of newborns. It typically appears on the forehead and upper eyelid, following the distribution of the first or second divisions of the trigeminal nerve. SWS is referred to as complete when both facial and leptomeningeal angiomas are present, and as incomplete when angiomas are isolated to either the face or the central nervous system, and has been classified according to Roach *et al* as follows: Type I which represents the classic complete syndrome, Type II with facial angiomas only, and Type III with isolated leptomeningeal angiomas^[4–6]^.

The leptomeningeal capillary-venous malformations (LCVMs), historically referred to as leptomeningeal angiomas, occur in 10–20% of cases when a characteristic facial port-wine stain is present. The intracerebral capillary-venous malformations usually occur ipsilateral to the port-wine stain, with the parietal and occipital lobes being the most commonly affected regions. Interestingly enough, any extension to the temporal lobes, such as the one in our case, is considered a somewhat unique finding. Neurological complications include developmental delays and seizures which are present in 75–90% of patients, and initially present as focal but often progress into generalized tonic-clonic seizures potentially due to variations in cortical perfusion patterns. They are also usually associated with a poorer prognosis if manifested within the first year of life^[5,7–9]^.

Acute hemiparesis may accompany seizure onset, typically affecting the contralateral side of the intracranial lesion due to chronic vascular insufficiency. Additionally, cognitive impairment is observed in approximately 60% of patients, with severe intellectual disability in a third of the cases^[4,5,8,9]^. In our case, the patient maintained an intact cognitive function despite the presence of a large leptomeningeal angioma extending into the right temporal lobe.

Ophthalmologic complications are considered relatively common in SWS, with glaucoma occurring in up to 50% of patients. Choroidal capillary-venous malformations, also known as choroidal hemangiomas, contribute to increased intraocular pressure, posing a significant risk of irreversible vision loss if left untreated^[4,6,8]^.

Despite its rarity, SWS presents a clinical challenge due to the variability in symptom severity and the progressive nature of neurological and ocular complications. Early diagnosis and appropriate management are crucial for improving patient outcomes.

Symptoms like acute headaches and hemiparesis in the context of SWS Type I warrant suspicion for “stroke like episodes”; however, the presence of meningeal irritation signs such as neck rigidity is highly suggestive of subarachnoid hemorrhage. These episodes are usually associated with microbleeds due to fragile leptomeningeal angiomas and can be confirmed with subsequent cerebrospinal fluid analysis^[8,9]^.

Our case illustrates a somewhat atypical presentation of a stroke-like episode associated with SWS, where a subarachnoid microhemorrhage caused significant meningeal irritation, leading to nuchal rigidity – a rare but important clinical finding in management. This could have been easily misinterpreted as a large hemorrhagic event if no further investigations were carried out^[8,9]^.

Another differential diagnosis is meningitis but was excluded as the patient had fever with meningeal irritation signs, and cerebrospinal fluid analysis was in line with stress-induced subarachnoid microhemorrhage.

Recurrent seizures, status epilepticus, and vascular events may exacerbate the cortical hypoperfusion. This phenomenon termed as “vascular steal” leads to increased cortical ischemia and results in gliosis and atrophy due to calcium and iron deposition, which further lowers the seizure threshold and worsens neurological outcomes^[4,6,9]^.

Among key differential diagnoses is Klippel–Trenaunay–Weber syndrome (KTWS) which shares features with SWS including port-wine stains of the extremities and face, but is distinguished by limb hemihypertrophy of both soft and bony tissues which is often associated with solid visceral tumors affecting the kidneys, adrenal glands, and the liver. While rare overlap cases exist, the absence of hemihypertrophy and the normal abdominal ultrasound findings were helped exclude KTWS^[6,9]^.

Beckwith–Wiedemann syndrome (BWS) was also considered as a differential diagnosis, due to its shared feature of a facial port-wine stain. However, it is distinguished by a triad of macroglossia, omphalocele, and organomegaly. A risk of visceral neoplasia is also noted. Patients with BWS also have a severe risk of hypoglycemia resulting from pancreatic islet-cell hyperplasia which is possibly life threatening. We excluded BWS with relative confidence based on the absence of its hallmark features, normal blood glucose levels, and normal abdominal ultrasound findings^[6,9]^.

Dyke–Davidoff–Masson syndrome, could be considered as a differential diagnosis as well, and it typically presents with partial or total cerebral hemiatrophy secondary to intrauterine or perinatal carotid artery infarction. Cerebral atrophy can also occur in SWS during infancy but was not seen in our patient^[6,9]^.

After initiation of age-appropriate anticonvulsant therapy and low-dose aspirin therapy, the patient achieved clinical stability over 2 years of follow-up, with complete recovery within the first 3 months and without any stroke-like episodes. His neurological examination revealed preserved strength and tone in all extremities, intact cranial nerve functions, and normal intraocular pressures (16 mmHg bilaterally). Visual acuity remained unaffected despite the diffuse choroidal hemangioma in the right eye^[4,6,9]^.

## Conclusion

In conclusion, this case report highlights the importance of considering Sturge–Weber syndrome (SWS) in children with a port-wine stain exhibiting seizures, and hemiparesis. Atypical manifestations such as meningeal irritation signs, including nuchal rigidity, should prompt further investigations to reach a conclusive diagnosis and help identify stroke-like episodes secondary to subarachnoid microhemorrhages due to extensive fragile leptomeningeal capillary-venous malformations like the one in our case, and despite preserved cognitive function. Timely diagnosis, effective seizure management, and stroke prevention strategies such as low-dose aspirin therapy are critical in improving outcomes for children with Sturge–Weber syndrome experiencing stroke-like episodes.

## Data Availability

Not applicable.

## References

[1] De La Torre AJ Luat AF Juhász C. A multidisciplinary consensus for clinical care and research needs for Sturge-Weber syndrome. Pediatr Neurol 2018;84:11–20. 29803545 10.1016/j.pediatrneurol.2018.04.005 PMC6317878

[2] Gagnier JJ Kienle G Altman DG. The care guidelines: consensus-based clinical case reporting guideline development. Glob Adv Health Med 2013;2:38–43. 10.7453/gahmj.2013.008 PMC3833570 24416692

[3] Agha RA Mathew G Rashid R. Transparency in the reporting of artificial intelligence–the TITAN guideline. PJS 2025;10:100082.

[4] Karn M Barma A Ojha L. Sturge Weber syndrome: a case report, Clin Case Rep 2024;12:e9452. 39355767 10.1002/ccr3.9452 PMC11442486

[5] Timilsina S Kunwor B Chhetri ST. Sturge-Weber syndrome: a case report. J Nepal Med Assoc 2023;61:890–92. 10.31729/jnma.8344 PMC10725224 38289732

[6] Balkuv E Isik N Aydin I. Sturge-Weber syndrome: a case report with persistent headache. Pan Afr Med J 2014;18:87. 25400854 10.11604/pamj.2014.18.87.3346 PMC4231313

[7] Naja K Kapciak A Górny J. Sturge-Weber syndrome: a comprehensive review of clinical features, optimized diagnosis and management strategies, Qual Sport 2024;36:56849.

[8] Parisi L Di Filippo T Grutta SL. Sturge-Weber syndrome: a report of 14 cases, Ment Illn 2013;5:7. 10.4081/mi.2013.e7 PMC4253385 25478131

[9] Mohamed S Sidow N Adam B. Undiagnosed epileptic case since childhood of Sturge-Weber syndrome: first case report from Somalia. Imcrj 2024;17:621–25. 10.2147/IMCRJ.S463858 PMC11215277 38952480

